# The High Density Polyethylene Composite with Recycled Radiation Cross-Linked Filler of rHDPEx

**DOI:** 10.3390/polym10121361

**Published:** 2018-12-08

**Authors:** David Manas, Miroslav Manas, Ales Mizera, Pavel Stoklasek, Jan Navratil, Stanislav Sehnalek, Pavel Drabek

**Affiliations:** 1 Faculty of Applied Informatics, Tomas Bata University in Zlin, CEBIA-Tech, Nad Stranemi 4511, 760 05 Zlin, Czech Republic; manas@utb.cz (D.M.); manas@fai.utb.cz (M.M.); pstoklasek@utb.cz (P.S.); sehnalek@fai.utb.cz (S.S.); pdrabek@utb.cz (P.D.); 2 SKODA AUTO a.s., tr. Vaclava Klementa 869, Mlada Boleslav II, 293 01 Mlada Boleslav, Czech Republic; jan.navratil@skoda-auto.cz

**Keywords:** High Density Polyethylene, radiation cross-linking, mechanical properties, structure, processability

## Abstract

This article discusses the possibilities of using radiation cross-linked high density polyethylene (HDPEx) acting as a filler in the original high density polyethylene (HDPE) matrix. The newly created composite is one of the possible answers to questions relating to the processing of radiation cross-linked thermoplastics. Radiation cross-linked networking is—nowadays, a commonly used technology that can significantly modify the properties of many types of thermoplastics. This paper describes the influence of the concentration of filler, in the form of grit or powder obtained by the grinding/milling of products/industrial waste from radiation cross-linked high density polyethylene (rHDPEx) on the mechanical and processing properties and the composite structure. It was determined that, by varying the concentration of the filler, it is possible to influence the mechanical behaviour of the composite. The mechanical properties of the new composite—measured at room temperature, are generally comparable or better than the same properties of the original thermoplastic. This creates very good assumptions for the effective and economically acceptable, processing of high density polyethylene (rHDPEx) waste. Its processability however, is limited; it can be processed by injection moulding up to 60 wt %.

## 1. Introduction

Plastic processing shows an increasing trend on an annual basis. Polymers—especially thermoplastics, are used in all industrial fields, and in a wide variety of applications—including packaging materials, household products, sports and leisure activities, medical products, and the automotive, aviation, and electro-technical and electronics fields. Concurrent with the production processes, large quantities of waste are generated and products at the end of their life-cycle accumulate. The quality of waste polymers depends on their origin, way of collection, and contamination by other types of polymers, or organic or inorganic pollution [1,2].

At present, more than one third of this waste is recycled, the same amount is landfilled, or used for energy recovery purposes [3]. Waste treatment methods vary considerably and involve several simple or complex operations like collecting, sorting cleaning and grinding them. Ground waste of a known source and composition can be used directly as additives to the original material or be processed into a granular form. The waste collection methods differ from country to country. Eventual contamination of this waste or plastic mixes of an unknown type can lead to problems in the course of their further processing [3,4,5,6,7,8,9,10,11,12,13,14,15]. Apart from material recycling, different chemical recycling methods are also used [4]. Recently, the issue of processing polymeric materials into synthetic gases has been addressed very intensively [16,17,18,19].

Along with the enormous developments in the processing of polymers field, so too has grown the significance of processing polymer waste. The latest data shows that the capacity for processing polymer waste is growing world-wide. In Europe, 31% of all extrusion capacity is recycled. Capacity is growing, especially in the processing of flexible plastic waste from households. Furthermore, there is a problem with the processing of multi-layer materials, which continue to remain un-recyclable [20,21]. Even the USA is seeing ever greater attention being paid to the processing of polymer wastes. The volume of rigid plastics collected for recycling in 2016 was nearly 4.5 times greater than that collected in 2017; while plastic of film recycling has grown for 12 consecutive years and more than doubled in comparison with 2005 [22]. Research has also been conducted into the prediction of the life-cycle of polymer films from polyolefins and biodegradable plastics [23,24].

Technologies that re-melt polymer waste are suitable for thermoplastics. This processing method; however, is not applicable to cross-linked thermoplastics, especially due to the impossibility of re-melting them. Modification of thermoplastics by cross-linking, in particular by radiation networking, is on the industrial scale, becoming increasingly widespread. The cross-linking process allows one to modify the properties of thermoplastics, in particular to improve their mechanical properties, to increase their temperature and chemical resistance, and thereby, to significantly increase the application potential of these materials. Some thermoplastics, like polyethylene (PE) for instance, cross-link without the addition of cross-linking agents. Others, for instance, polypropylene (PP) or polyamides (PA), require cross-linking agents in order to cross-link [25,26,27].

In line with the increasing share of such modified thermoplastics, so too does the share of waste generated by its own cross-linking and, in particular, the products of cross-linked thermoplastics at the end of their lifetime. Radiation cross-linking allows significant improvements in some properties, but the possibility of recycling in traditional ways is ruled out. Radiated cross-linked polymers lose their ability due to the creation of spatial networks in the original thermoplastics to be re-meltable [28,29,30,31,32,33,34]. Therefore, the processing/recycling of radiation cross-linked thermoplastics is much more complicated when compared to the original polymer; so far, there are no effective ways to recycle these materials on an industrial scale. Research on radiation-cross-linked HDPEx on the properties of HDPEx-LDPE composites has shown that the composite formed in this way is processable by conventional techniques used in the processing of thermoplastics, in particular by injection moulding technology. A higher content of HDPEx filler in the LDPE matrix makes composite processability worse. Most of the mechanical properties of the composite are significantly better than the mechanical properties of the matrix itself [32,33,34]. The use of recycled cross-linked rHDPEx as a filler in low density polyethylene (LDPE) is described in the work [35].

This paper presents a study of the possibility of cross-linked high density polyethylene (rHDPEx) waste recycling as a filler in original (virgin) HDPE. There is described the possibility of creating a new type of high density polyethylene (HDPE) composite with a high density polyethylene radiation cross-linked filler (rHDPEx) and the influence of content of HDPEx filler on its characteristics and structure.

## 2. Materials and Methods

### 2.1. Material

The wide availability of rHDPEx was the main reason for the use of this polymer in the research presented herein. This material was supplied in the form of heating pipes used for underfloor heating systems. The pipes were crushed using a MASKIN AB RAPIS S-33010, low-speed knife mill, screen-size 5 mm and ground using a Condux toothed disc mill. To obtain crushed or powdered material for further processing, the particles with dimension higher than 1 mm were separated from the powder using a 1 mm screen. High density polyethylene (HDPE—Tipelin 6300B, Slovnaft, Bratislava, Slovakia) was chosen as the polymer matrix regarding its ease of processability, low price and wide availability. HDPE granulate was used as the polymer matrix and rHDPEx in grit or powder states were used as the filler.

### 2.2. Irradiation

The irradiation of used HDPE pipes originally intended for use in underfloor heating systems was originally carried out at the BGS Beta-Gamma-Service company, Germany premises. Electron beam radiation, at the dose of 165 kGy, and energy of 10 MeV was used for their irradiation. The cross-linking of used HDPE was realised without the use of a cross-linking agent. Gel content measurement and a dosimeter were used to determine and find proof of correctness. The correct radiation dose was checked by a nylon FTN 60-00 dosimeter; and the analysis was performed using a Genessis 5 spectrophotometer in correspondence with the ASTM 51261 standard [36]. Gel content was measured according to the ASTM D7567 standard [37], which is determined by means of its solvent extraction with xylene. The dose of 165 kGy corresponds to 60% gel content.

### 2.3. Specimen Preparation

**Figure 1:**
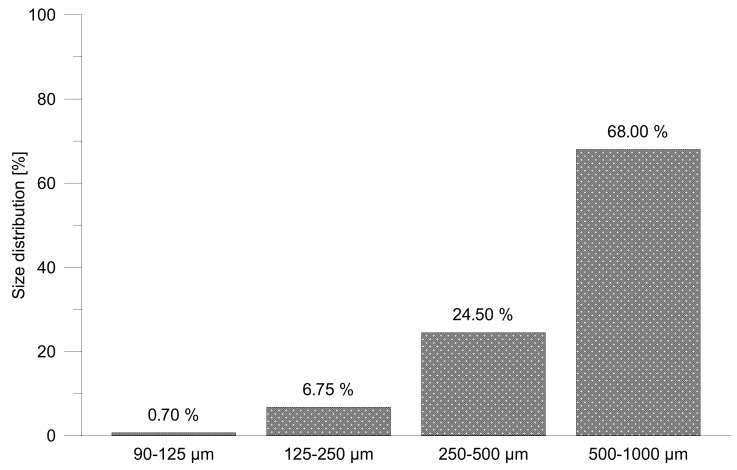
Powder—size distribution.

**Figure 2:**
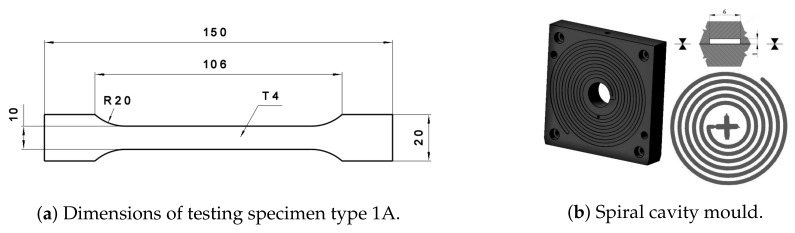
Test speciment.

From crushed and powdered material, two types of mixtures which differ from each other by filler state (grit or powder rHDPEx) were prepared: mixture A (HDPE granulate + rHDPEx grit) and mixture B (HDPE granulate + rHDPEx powder). Crushed (particle sizes of 1–5 mm) or powdered rHDPEx was mixed together with virgin HDPE granulate in a laboratory fluid mixer. A size analysis was performed using Retsch AS 200 Basic equipment for the determination of particle size distribution (Figure 1). Dimensions of 68% particles are in interval 0.5 to 1.0 mm and only less than 0.05% of the particles are smaller than 0.09 mm. From this mixture, in concentrations ranging from 10 to 60 wt % of rHDPEx in virgin HDPE, Type 1A specimens for all tests according to the ISO 527 standard [38] were prepared by injection moulding using an Arburg Allrounder 470H injection moulding machine. The dimensions of specimens are given in Figure 2a and the processing conditions are given in Table 1.

15 specimens were prepared for each testing method and statistical evaluation was realised in the TestXpert II, MS Excel 2016 and MiniTab 16 programs. Arithmetic mean and standard deviation are used in all figures.

### 2.4. Polymer Mixture Fluidity

A modified spiral test was performed using a spiral cavity mould [39] with a spiral length of 2000 mm; the shape of the cavity is rectangular, its dimensions were 6 mm × 1 mm (Figure 2b). An Arburg Allrounder 470H injection moulding machine was used for the preparation of test spirals. The injection moulding processing conditions are the same as in the “Specimen preparation” paragraph (Table 1).

### 2.5. Tensile Test

A ZWICK 1456 tensile machine was used for the estimation of tensile behaviour. Measurements were carried out according to the ISO 527 standard [38]—at ambient (23 ${}^{\circ }$C) and elevated (80 ${}^{\circ }$C) temperatures; crosshead speed: 50 mm/min. The E-modulus and ultimate tensile strength were all evaluated from these measurements. Conditioning was taken for 5 days in temperature of 23 ${}^{\circ }$C and relative humidity of 50%.

### 2.6. Shore D Hardness (ShD)

An OMAG AFRI ART 13 Shore D hardness tester was used for hardness testing in accordance with the ISO 868 standard [40], therefore holding time was 15 s.

### 2.7. Vicat Softening Temperature

An HDT 6 Vicat CEAST type 6921 device was used to measure the Vicat softening temperature (VST). The test was carried out in accordance with the CSN EN ISO 306/A50 standard [41]. The test specimens were heated in an oil bath, at a heating rate of 50 ${}^{\circ }$C/h. The VST was automatically detected at a penetration point of 1 mm${}^{2}$ to 1 mm depth of the test body; the loading force was 10 N.

### 2.8. Structural Analysis

A LEICA RM2255 microtome was used for the preparation of samples, (thickness of 35 μm) and an OLYMPUS BX41 microscope was used for the structural analysis. Equally, the structure was investigated on the fracture area with nitrogen-cooled bodies using a JEOL 7500F scanning electron microscope.

## 3. Results and Discussion

Two types of mixtures A (HDPE + rHDPEx grit) and B (HDPE + rHDPEx powder) were prepared and tested. The basic processing characteristics of the polymer blend with different doses of filler (rHDPEx) were measured and changes in the mechanical properties of the studied HDPE + rHDPEx composite were monitored.

**Figure 3:**
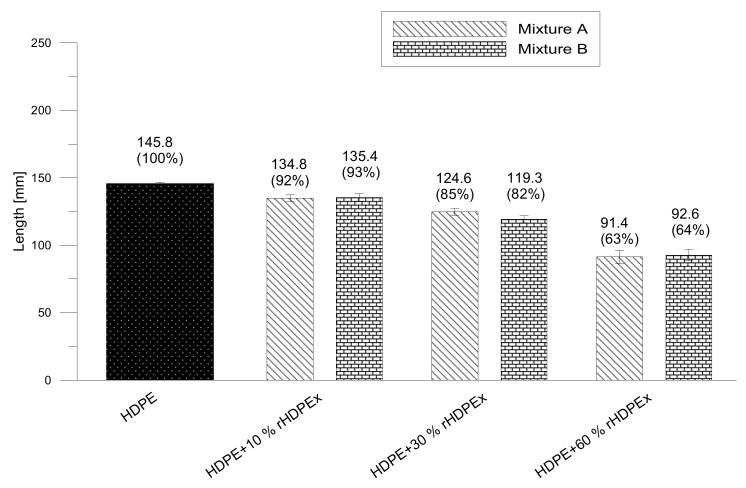
Polymer fluidity comparison—mixtures A, B.

The basic processing properties of the polymers and their possible mixtures are usually characterised by the melt flow index (MFI). Due to the rHDPEx particle size and the impossibility of re-melting, MFI measurements could not be performed. A flow modified spiral test was performed to assess flow properties because of possibility of using a high injection rate and filling pressure which caused that melted HDPE and softened rHDPEx pass through injection gate with dimensions 6 × 1 mm. From the measured data, it is clear that the filler content (rHDPEx) significantly influences the polymer melt viscosity. The length of the spiral arrest decreased as the filler content increased. The flow properties of the A and B mixtures deteriorated at the filler content of 10 wt %, where the spiral length was 8% lower and decreased steadily with a further increase in the proportion of the filler. At the maximum dose rHDPEx of 60 wt % in the original HDPE led to a drop in the spiral length by up to 37% as compared to HDPE. This filler content (60 wt %), appears to be the boundary limit, since, with a higher rHDPEx content it was no longer possible to process the mixture. A similar trend can be observed for mixture B (HDPE + rHDPEx powder) with very small differences between the measured values of both mixtures (Figure 3).

**Figure 4:**
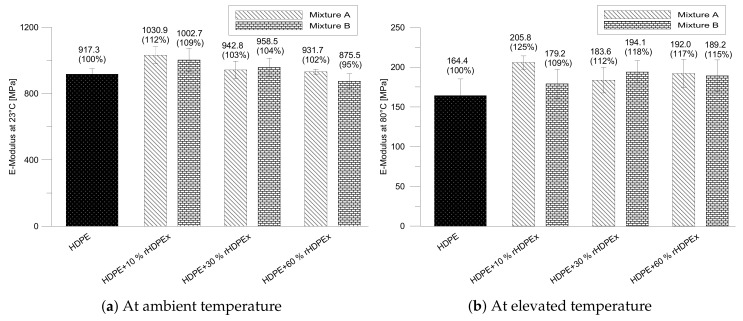
E-modulus of mixtures A, B.

The measurements made show that mechanical properties are also influenced by the filler content. Dose 10 wt % rHDPEx causes the E-modulus (modulus of elasticity) to increase by up to 12% (mixture A) and 9% (mixture B) in both tested mixtures. The greatest decrease in the E-modulus was recorded at the highest concentration of filler (HDPEx) in the form of a powder. Since a slight decrease in the E-modulus occurred at the same concentration in the A mixture, these changes can be attributed in particular to worsening of the processability/miscibility of the mixture at the highest concentrations of the filler. As the filler concentration increases, the E-modulus gradually decreases slightly. With regard to the measurement error, it can be stated that the E-modulus of both test mixtures, measured at room temperature (23 ${}^{\circ }$C) at a filler concentration of 30 and 60 wt % is approximately the same as the E-modulus of the matrix itself (virgin HDPE), which is very important from the usability in working practise perspective (Figure 4a). The E-modulus of mixture A at elevated temperature (80 ${}^{\circ }$C) shows significant changes with a maximum increase of 25% at a filler content of 10 wt %. Mixture B results in a gradual increase in the value of the E-modulus and at the maximum filler content—(60 wt %). The E-modulus at 60 wt % is approximately the same as that of A (Figure 4b). In both cases, the E-modulus values measured in the course of increasing the temperature are higher across the full range of filler doses are higher by 9 to 25% than the matrix (HDPE).

**Figure 5:**
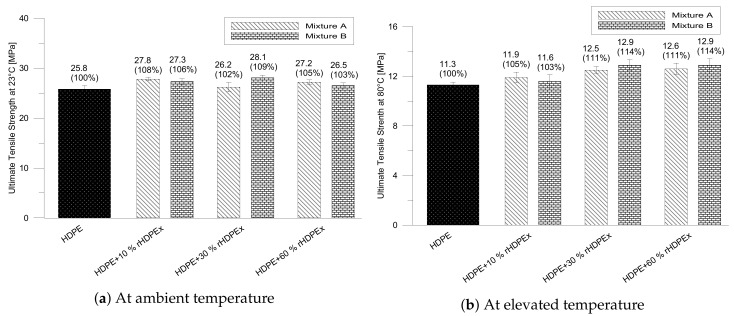
Ultimate tensile strength of mixtures A, B.

For both mixtures, the strength (measured at 23 ${}^{\circ }$C) increased slightly, in line with the increasing charge of the filler with a maximum increase of 8% for mixture A and a dose of 10 wt % filler; or respectively, 9% for mixture B, at a dose of 30 wt % filler. At the maximum filler content (60 wt %), tensile strength is slightly higher than the tensile strength of the matrix (HDPE) itself (Figure 5a). The tensile strength of the two test mixtures, measured at an elevated temperature (80 ${}^{\circ }$C) is more pronounced. Along with the increasing filler dose, the strength limit gradually increases with the maximum of 11% for mixture A; and respectively, of 14% for B, at the maximum filler content (60 wt %). The tensile strength at elevated temperature is higher than the tensile strength of the matrix (Figure 5b).

**Figure 6:**
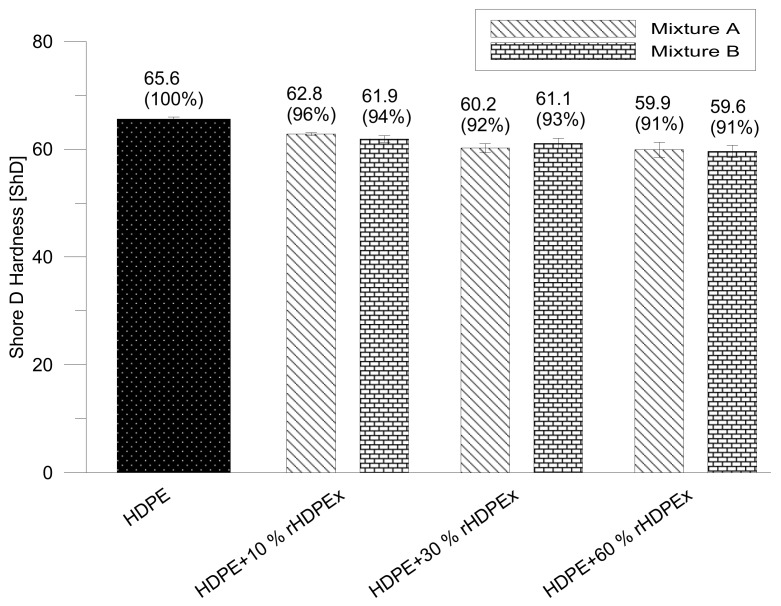
Shore D hardness—mixtures A, B.

The Shore D hardness of both of the tested mixtures with the filler dose declined slowly. The minimum hardness (9% lower than the matrix hardness) was measured for a mixture with a filler dose of 60 wt % (Figure 6).

**Figure 7:**
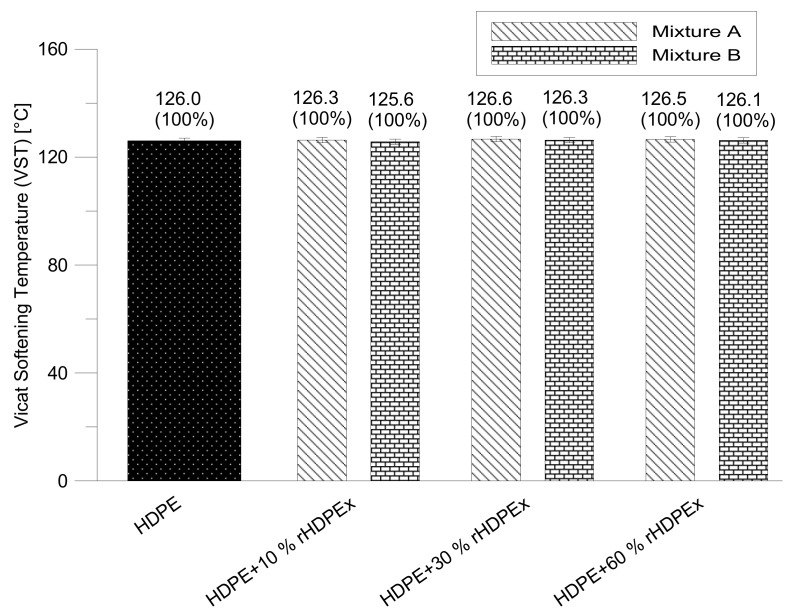
Vicat testing—mixture A, B.

In the course of measuring the VST, no changes were recorded for both mixtures when comparing HDPE to HDPEx (Figure 7).

**Figure 8:**
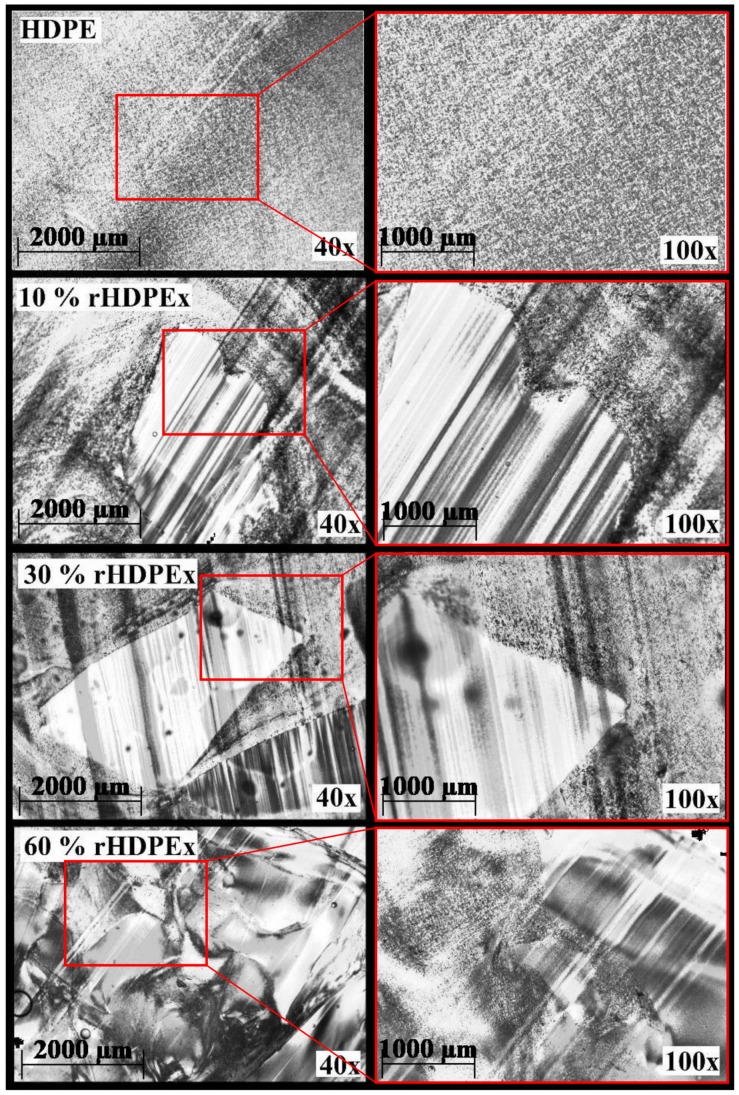
Microtome cuts of mixture A.

**Figure 9:**
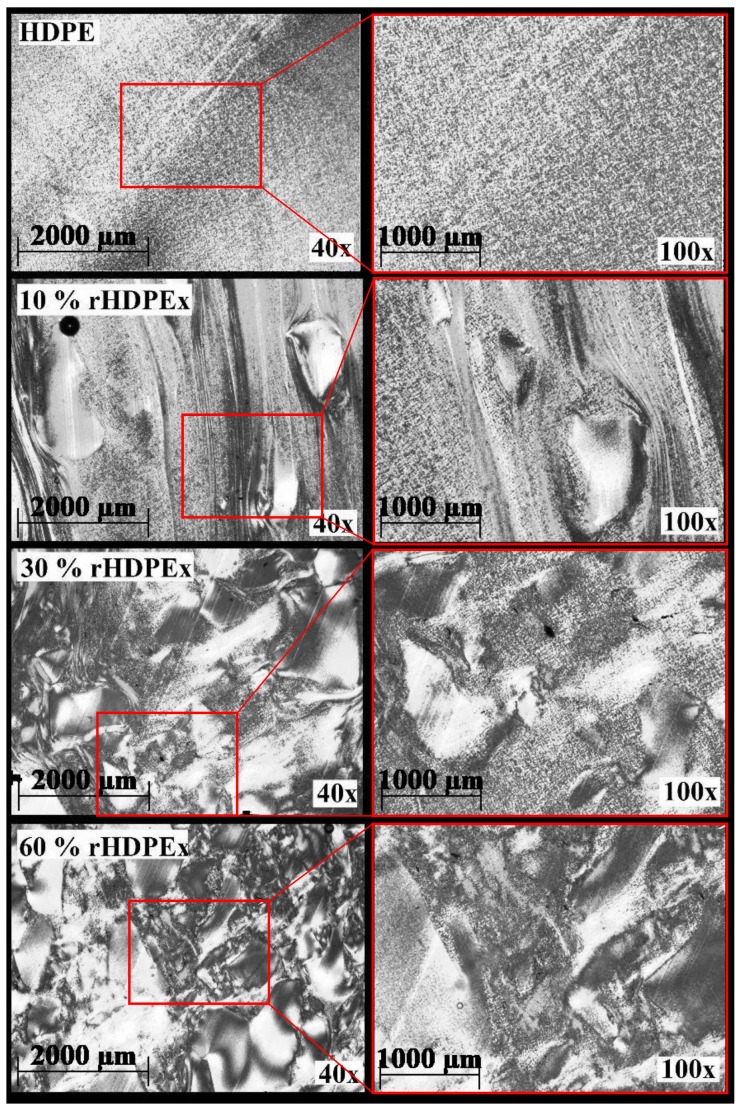
Microtome cuts of mixture B.

Figure 8 shows the microtome cuts of mixture A. At all concentrations, the perfect fit of the rHDPEx filler material can be observed, which is coated with HDPE without visible damage even at a concentration of 60 wt % rHDPEx filler. Similar features can be seen in Figure 9, where rHDPEx powder was added instead of grit at the same filler concentration. Figure 9, shows better dispersion as compared to the grit. Structural tests confirmed these findings. However, the mechanical properties show that the additional grinding process is economically disadvantageous when the properties remain similar to those of the less-costly crushing process.

**Figure 10:**
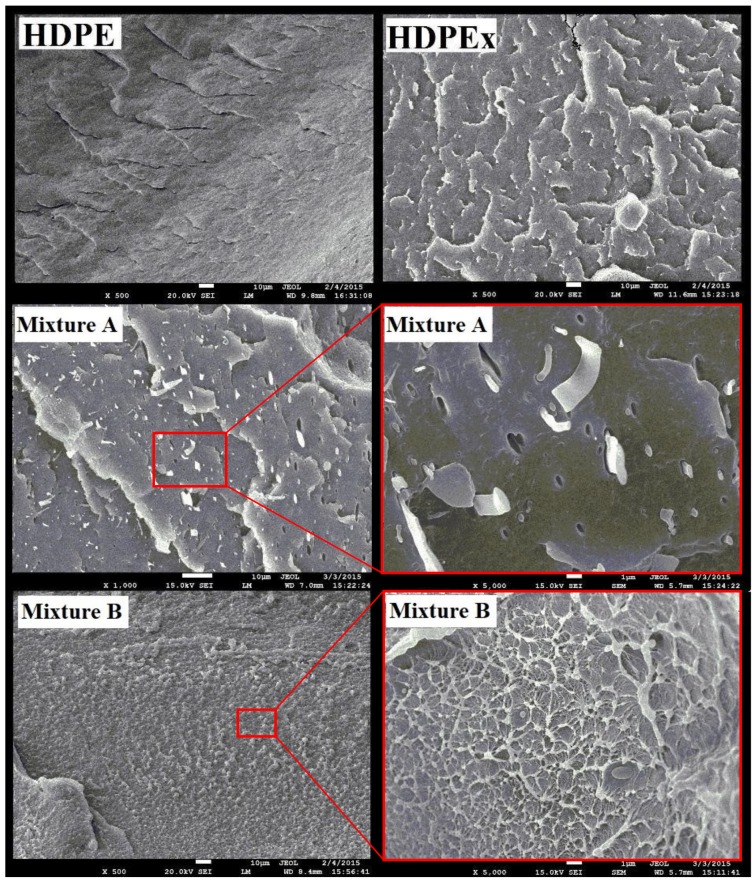
SEM—fracture surface structure.

Lastly, the SEM of the fracture surface—(low-temperature—liquid N${}_{2}$) was used—as shown in Figure 10. The orientation of the lamellae can be observed on the HDPE fracture surface. In contrast, HDPEx has a cross-linked structure, which occurs on the fracture surface by the tearing of bonds. When HDPEx is crushed or ground, there is a splitting of particles with a rough surface that have a larger contact surface than crushed or milled HDPE. Because HDPEx is no longer meltable, it only softens—due to the large interface, and an HDPE matrix with the rHDPEx filler grit or powder is perfectly bonded. Although the mechanical properties of Mixtures A and B are similar, the fracture surface appears different. For Mixture A, the region of the tearing of the cross-linked fillers, this can be seen in Figure 10. On the other hand, Mixture B produced a perfect mix of rHDPEx powder with the HDPE matrix. The non-meltable rHDPEx powder prevented the formation of a lamellar structure and resulted in a cellular structure, which had similar mechanical properties to the rHDPEx grit filler. This confirms that when processing both composites, the HDPE matrix with rHDPEx filler was bonded on the interphase interface. It is likely that the melted HDPE matrix bonds with the partially melted surface of the cross-linked rHDPEx filler, (gel content 60%). The structure of both mixtures confirms the very good and strong adhesion between the HDPE matrix and the filler—(rHDPEx). This visible interface between the matrix and the filler demonstrates that rHDPEx acts as a filler in both mixtures—A and B. From the results, it can be seen that recycled high-density polyethylene can be processed as a filler in virgin HDPE. The principle used is very simple and allows easy processing of the cross-linked polymer waste.

## 4. Conclusions

The aim of this paper was to describe the possibility of using recycled high-density polyethylene (rHDPEx). For the purposes of this study, waste originating from the processing of underfloor heating pipes was used to examine the effect of the different sizes (of grit and powder) of the waste, and its concentration in the matrix on the mechanical properties and the ability to process the prepared mixtures.

It was found that the addition of rHDPEx to virgin HDPE did not deteriorate the mechanical properties of the composite; on the contrary, in some cases—improvements occurred. An HDPE-based composite with a filler of recycled radiation cross-linked rHDPEx may be processed by injection-moulding up to 60 wt %. At this maximum dose, only a slight decrease in the flow-ability in the spiral, (about 35%), occurred. On the one hand, higher concentrations caused problems with product-compactness; while, on the other hand, it was necessary to increase the injection pressure to avoid clogging the injection sprue or prematurely freezing of the injection gate. No delamination at the HDPE and rHDPEx phase interface was observed for all concentrations tested.

The following results can be highlighted:

- The measured properties of the new composite change only slightly. The composite properties are superior to those of the virgin HDPE.
- It is possible to use the rHDPEx filler in the form of grit or powder.
- Both mixtures—HDPE with rHDPEx as filler, can be processed by injection-moulding up to a filler content of 60 wt %.
- One can say that a new way of waste-processing from radiation cross-linked polymer (rHDPEx) has been found.

The proposed processing method is very simple and involves only a few steps—(crushing or grinding of rHDPEx waste), and requires relatively small investments and low energy costs for the preparation of the given filler (rHDPEx). The method of using radiation cross-linked rHDPEx can be extended by further steps like the preparation of the given granulate mixture. However, the studies and tests that were conducted show that it is possible to dispense the powder directly into the HDPE matrix, thereby achieving very good properties for the new composite that are fully comparable with the original HDPE and without any unnecessary increase in recycling costs. The proposed method opens the way to an economically interesting manner of processing otherwise difficult to apply polymer waste.

The expansion of polymer waste from irradiated products is expected in the future. The radiation cross-linking technique has become the common way of modifying thermoplastic properties; not only HDPE but also engineering plastics like PP, PA, PBT etc. are irradiated. In a few years time their end-of-life period will finish, so the question is how to recycle this waste?

## Figures and Tables

**Table 1:** Injection moulding processing parameters.

**ARBURG ALLROUNDER 470H**		
Injection Velocity	60	mm s${}^{-1}$
Injection Pressure	80 (100 ${}^{1}$)	MPa
Cooling Time	20	s
Mould Temperature	40	${}^{\circ }$C
Holding Time	25	s
Holding Pressure	60 (85 ${}^{1}$)	MPa
**Temperature of Plasticizing Unit Zones**		
Temperature under the Hopper	60	${}^{\circ }$C
Temperature Zone 1	200	${}^{\circ }$C
Temperature Zone 2	215	${}^{\circ }$C
Temperature Zone 3	220	${}^{\circ }$C
Temperature Zone 4	235	${}^{\circ }$C
Temperature Zone 5	250	${}^{\circ }$C

${}^{1}$ used at the highest concentration of filler (60 wt %).
